# Effect of silica-coated magnetic nanoparticles on rigidity sensing of human embryonic kidney cells

**DOI:** 10.1186/s12951-020-00730-2

**Published:** 2020-11-18

**Authors:** Abdurazak Aman Ketebo, Tae Hwan Shin, Myeongjun Jun, Gwang Lee, Sungsu Park

**Affiliations:** 1 School of Mechanical Engineering, Sungkyunkwan University, Suwon, 16419 Korea; 2 Department of Physiology, Ajou University School of Medicine, Suwon, 16499 Korea

**Keywords:** Lamellipodia, Filopodia, Rigidity sensing, Silica-coated magnetic nanoparticles, Traction force

## Abstract

**Background:**

Nanoparticles (NPs) can enter cells and cause cellular dysfunction. For example, reactive oxygen species generated by NPs can damage the cytoskeleton and impair cellular adhesion properties. Previously, we reported that cell spreading and protrusion structures such as lamellipodia and filopodia was reduced when cells are treated with silica-coated magnetic nanoparticles incorporating rhodamine B isothiocyanate (MNPs@SiO_2_(RITC)), even at 0.1 μg/μL. These protruded structures are involved in a cell’s rigidity sensing, but how these NPs affect rigidity sensing is unknown.

**Results:**

Here, we report that the rigidity sensing of human embryonic kidney (HEK293) cells was impaired even at 0.1 μg/μL of MNPs@SiO_2_(RITC). At this concentration, cells were unable to discern the stiffness difference between soft (5 kPa) and rigid (2 MPa) flat surfaces. The impairment of rigidity sensing was further supported by observing the disappearance of locally contracted elastomeric submicron pillars (900 nm in diameter, 2 μm in height, 24.21 nN/μm in stiffness *k*) under MNPs@SiO_2_(RITC) treated cells. A decrease in the phosphorylation of paxillin, which is involved in focal adhesion dynamics, may cause cells to be insensitive to stiffness differences when they are treated with MNPs@SiO_2_(RITC).

**Conclusions:**

Our results suggest that NPs may impair the rigidity sensing of cells even at low concentrations, thereby affecting cell adhesion and spreading. 
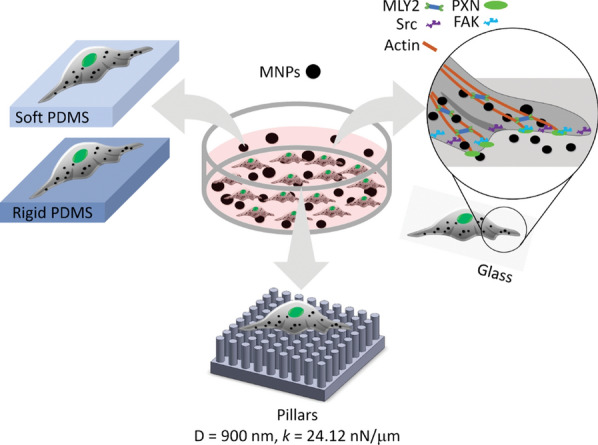

## Background

In recent years, the use of nanoparticles (NPs) has been rapidly growing in medical research, especially for diagnostic and therapeutic purposes. The size of NPs enables them to enter cells and accumulate, causing cellular dysfunction [1–4]. Moreover, because of their high surface-to-volume ratios, NPs are highly reactive and potentially have side effects, like generating reactive oxygen species (ROS), compared to bulk material [5–7]. ROS damages cell membranes, cytoskeletons, etc. [8–12].

Magnetic nanoparticles (MNPs) are widely used in diagnostics and as biosensors in biotechnology and biomedicine [13, 14]. To reduce the adverse effect of MNPs, they are coated with biocompatible components such as polyethylenimine, polysaccharide, and silica [15–18]. Among these MNPs, silica-coated magnetic nanoparticles incorporating rhodamine B isothiocyanate (MNPs@SiO_2_(RITC)) composed of silica shells and MNP cores in the range from 1 to 10 μg/μl are used for cell labeling [18], hyperthermia [19] and magnetic resonance imaging (MRI) [20]. MNPs@SiO_2_(RITC) have been evaluated to be non-toxic by conventional methods for assessment of toxicity [21–23]. It was reported that MNPs@SiO_2_(RITC) did not cause apparent toxicity in mice when administrated into them at the concentration of 25 mg to 100 mg/kg [21]. Unlike these results, we reported that MNPs@SiO_2_(RITC) induce the production ROS, that leads to ER stress, decreased proteasome activity, and altered cellular metabolism [4, 9, 24], suggesting that careful studies are required before the applications of MNPs@SiO_2_(RITC) in vivo.

During their initial contact, cells sense extracellular matrix (ECM) rigidity, in a phenomenon called rigidity sensing. Rigidity sensing is required for cells to translate the mechanical properties of the ECM into biochemical signals that can regulate the genes and proteins of the cell [25–27]. Biochemical signaling is involved in determining cell behaviors and fates, such as cell differentiation, migration, apoptosis, proliferation, and tissue development [28–30]. NPs disrupt the cytoskeleton, affecting focal adhesion (FA) proteins and their subsequent adhesion [12], which are initiated beneath lamellipodia (branched actin filaments) and filopodia (finger-like protrusions) as focal complexes [31]. Previously, we reported that the formation of lamellipodia and filopodia were inhibited at 0.1 and 1 μg/μL of MNPs@SiO_2_(RITC) [23]. However, it is still unknown how these NPs affect the cell's ability to sense the stiffness of the ECM.

Conventionally, cell rigidity sensing is studied by observing changes in cell morphology using flat polydimethylsiloxane (PDMS) surfaces with stiffness of 5 kPa (soft) and 2 MPa (rigid). Cells respond to a rigid surface by forming polarized shapes with a large FA area [32]. Furthermore, polarized cells often show filopodia that probe the substrate rigidity before spreading [33]. Recently, rigidity sensing has been studied by measuring local contractions on elastomeric submicron pillars [34, 35]. Local contractions are detected by observing the bending of adjacent pillars toward each other when cells were seeded on the pillars [36].

**Fig. 1:**
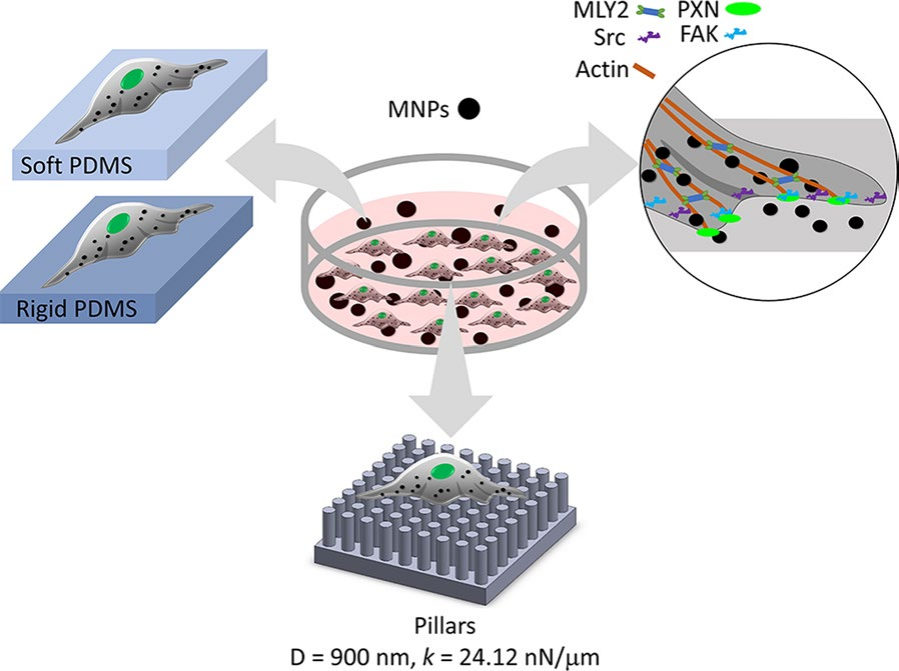
Schematic diagram showing the methods used to study the effect of MNPs@SiO_2_(RITC) on rigidity sensing of HEK293 cells

In this report, we describe the effect of MNPs@SiO_2_(RITC) on the mechanobiological aspects of human embryonic kidney cells (HEK293) using soft (5 kPa) and rigid (2 MPa) flat PDMS surfaces as well as elastomeric submicron pillars (900 nm in diameter, 2 μm in height, 24.21 nN/μm in stiffness *k*) (Fig. 1). To understand how the treatment of MNPs@SiO_2_(RITC) impaired the rigidity sensing, western blotting was used to assess alterations in the phosphorylation of the cytoskeletal proteins.

## Results

### Effects of MNPs@SiO_2_(RITC) on the morphology of cells on cover slips

**Fig. 2:**
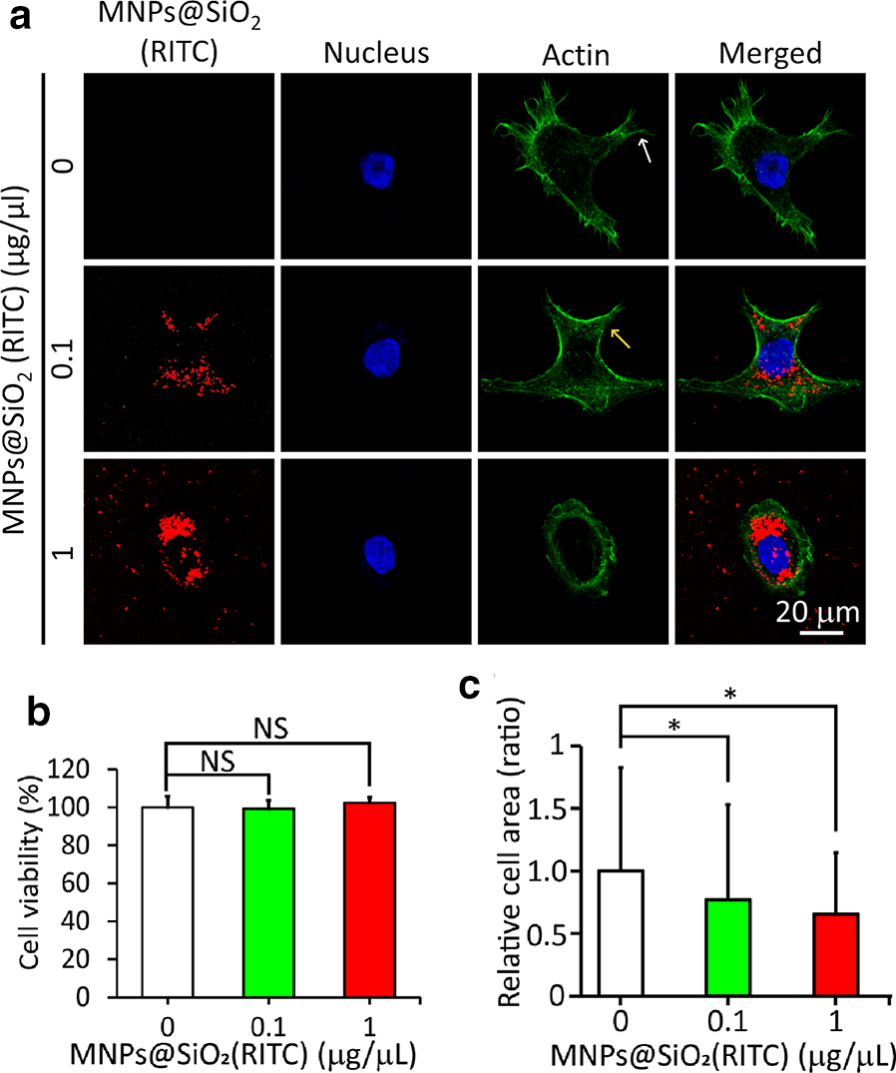
Cell viability, spreading and lamellipodium and filopodium formation in HEK293 cells at different concentrations (0–1 μg/μL) of MNPs@SiO_2_(RITC). **a** Fluorescence images of cells taken using confocal laser scanning microscopy (CLSM) (LSM710). Green, F-actin; red, MNPs@SiO2(RITC); blue, Hoechst 33342. Scale bar = 20 µm. Lamellipodia and filopodia were indicated by yellow and white arrows. **b** Viability of cells at 12 h after MNPs@SiO_2_(RITC) treatment. The experiment is performed in triplicate samples. **c** Relative area (ratio) of cells on cover slips were taken after 12-h incubation with and without MNPs@SiO_2_(RITC). The relative area was calculated by dividing the cell area of the treated cells by the cell area of the untreated cells. Data represent the mean ± standard deviation (SD). Cell number (n) > 400. Student’s t-test. * P < 0.05. NS: not significant

Viability of cells was evaluated after treating them with 0.1 or 1 μg/μL of MNPs@SiO_2_(RITC) for 12 h (Fig. 2b). There was no significant difference between the untreated and treated cells with MNPs@SiO_2_(RITC) at 0.1 and 1 μg/μL. MNPs@SiO_2_(RITC) were found in the cells at both concentrations in a concentration-dependent manner (Fig. 2a). The untreated cells spread with lamellipodia (indicated by yellow arrows) and filopodia (indicated by white arrows). At 0.1 μg/μL, the cells lost filopodia (Fig. 2a), and their relative cell area was significantly smaller than that of the untreated cells (Fig. 2c). At 1 μg/μL, the cells became round and did not display any extended structures, which may explain why their relative cell area was smaller than that of the untreated cells (Fig. 2c).

### Effect of MNPs@SiO_2_(RITC) on cell spreading and polarization on soft and rigid surfaces

**Fig. 3:**
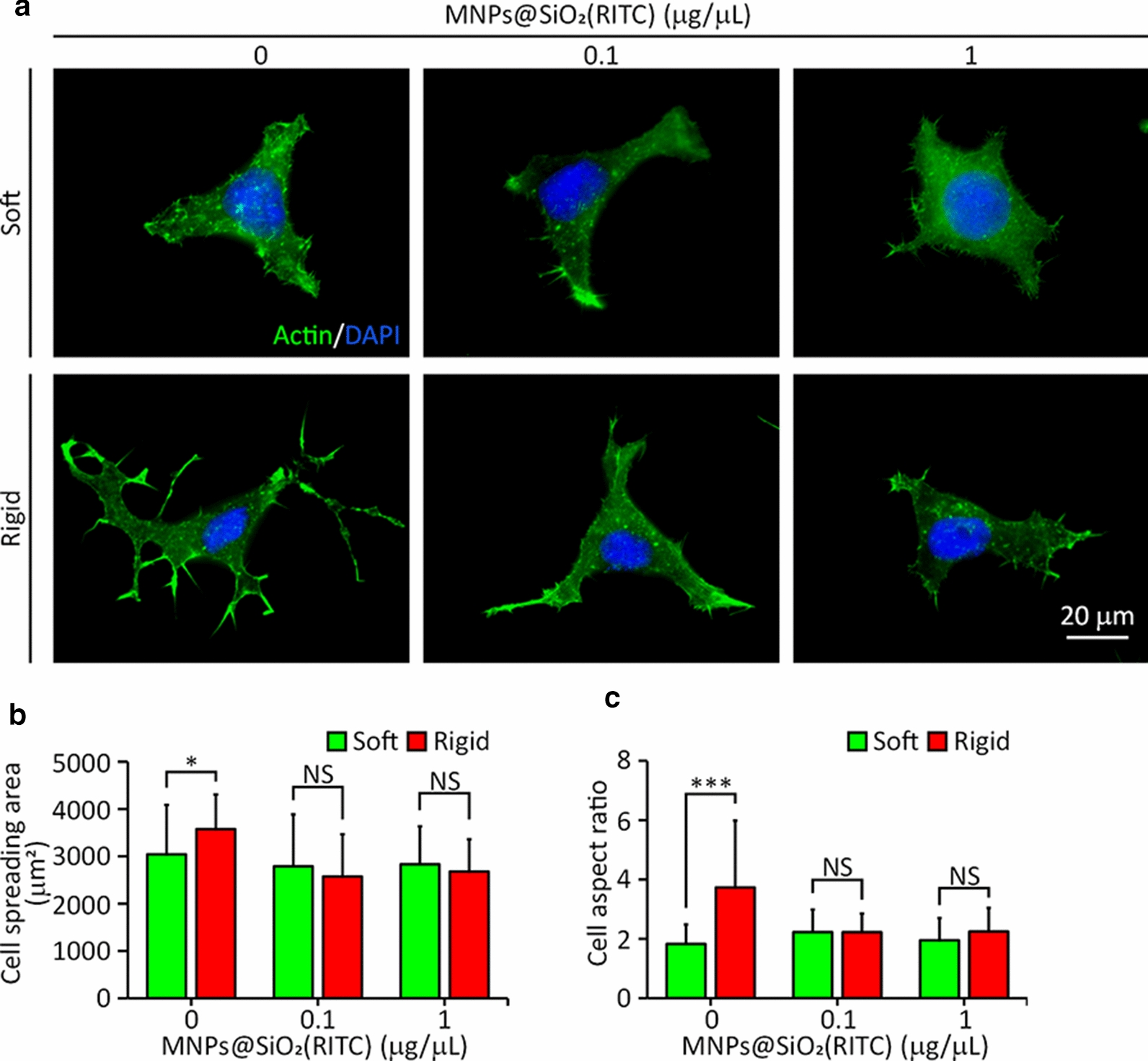
Spreading and aspect ratio of HEK293 cells on soft (5 kPa) and rigid (2 MPa) surfaces after treatment with different concentrations (0, 0.1, or 1 μg/μL) of MNPs@SiO_2_(RITC). **a** Fluorescence images of cells with actin (green) and DAPI (blue) staining. **b** Cell spreading area and **c** aspect ratio. (n = 30 cells). NS: not significant. * P < 0.05, *** P < 0.001. Student’s t-test

Since the extent of cell spreading depends on the substrate rigidity [37], cells were incubated on soft and rigid surfaces prepared with PDMS. As shown in Fig. 3a, the untreated cells on the soft surfaces (5 kPa) spread less without filopodia than those on the rigid surfaces (2 MPa). It should be pointed out that the total apical cell surface area on the soft surfaces could be underestimated by the projected area because they are usually thicker than those grown on the rigid surface [38]. This result showed that the cells could distinguish the rigidity difference between the surfaces. Upon treatment with 0.1 or 1 μg/μL of MNPs@SiO_2_(RITC), the morphologies of cells on the soft and rigid surfaces looked similar because filopodium formation and cell spreading on both surfaces surface were inhibited.

This inhibitory effect was further analyzed by measuring the cell spreading area and aspect ratio on both surfaces. Without treatment, the cell spreading area on soft surfaces (3042 μm^2^) was significantly lower than that on rigid surfaces (3575 μm^2^) (Fig. 3b). However, with the treatment of MNPs@SiO_2_(RITC) at either 0.1 or 1 μg/μL, the cell spreading areas on both surfaces decreased to about 3000 μm^2^, indicating that the treatment caused cell shrinkage. These results showed that cell spreading on rigid surfaces was inhibited by MNPs@SiO_2_(RITC).

A similar trend was observed for the cell aspect ratio. Without MNPs@SiO_2_(RITC) treatment, the cell aspect ratio on the rigid surface was ~ 4. Upon treatment, the cell aspect ratio on the rigid surface decreased to a similar level as the cell aspect ratio on the soft surface (~ 2) (Fig. 3c). This result showed that the cells were not polarized due to MNPs@SiO_2_(RITC) treatment on rigid surfaces. On soft surface, cells tend to have a round shape. Taken together, it is suggested that the ability of cells to distinguish soft from rigid surfaces is greatly impaired by MNPs@SiO_2_(RITC).

### Effect of MNPs@SiO_2_(RITC) on F-actin distribution on soft and rigid surfaces.

**Fig. 4:**
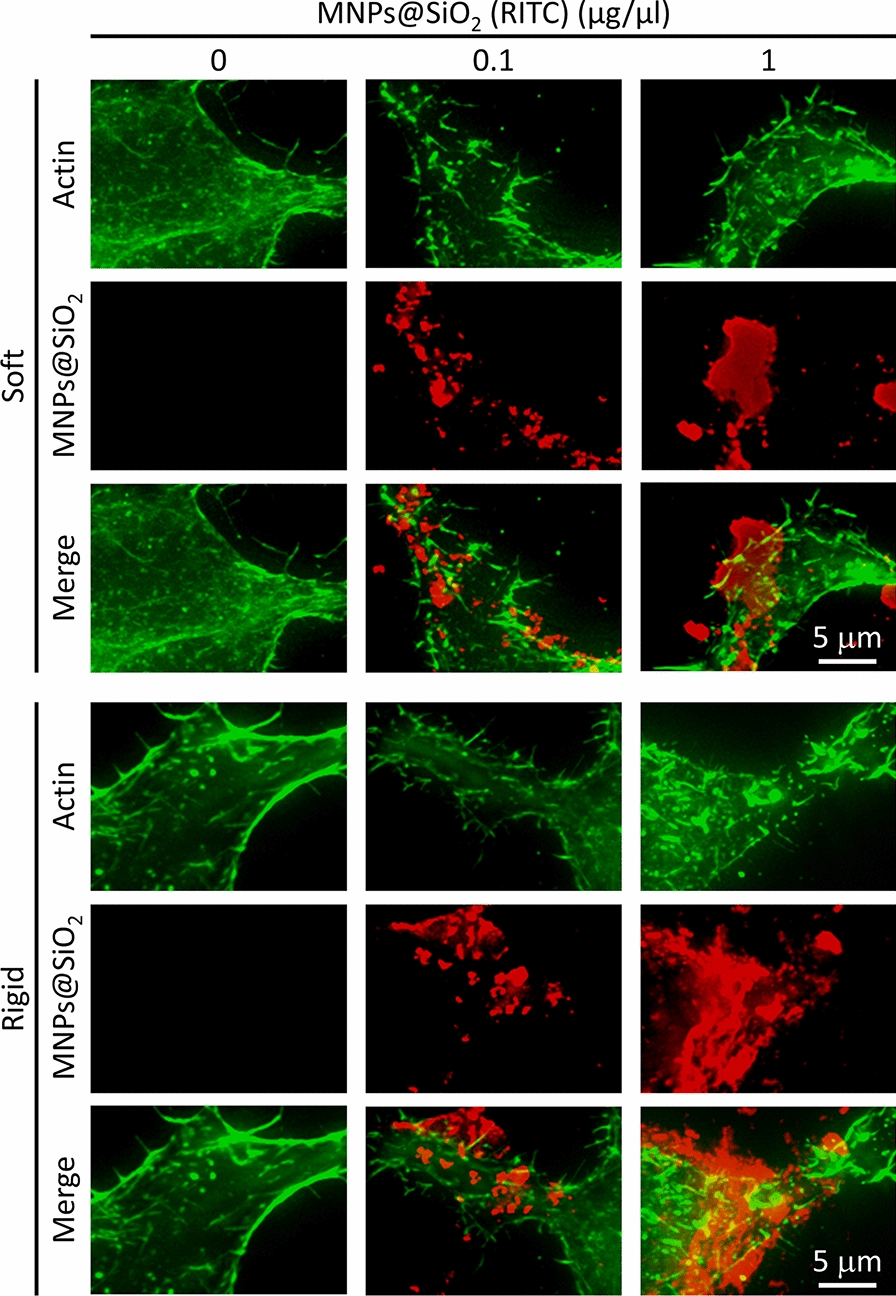
Staining of actin filaments on HEK293 cells on soft (5 kPa) and rigid (2 MPa) surfaces after treatment with different concentrations (0–1 μg/μL) of MNPs@SiO_2_(RITC) for 12 h. For actin labeling, cells were incubated with Alexa Fluor 488-conjugated phalloidin (Molecular Probe, Eugene, OR, USA), (1:200) diluted in PBS, for 1 h at room temperature

To understand how cell spreading on the rigid surfaces was inhibited by MNPs@SiO_2_(RITC), we investigated the effect of MNPs@SiO_2_(RITC) on the actin structure of cells. Cells were grown on soft and rigid surfaces with and without MNPs@SiO_2_(RITC) for 12 h and both actin filaments and MNPs@SiO_2_(RITC) were visualized as shown in Fig. 4. The untreated cells on soft surfaces displayed thin actin fibers around their periphery, while those on rigid surface displayed thick fibers. This result showed that the thickness of actin stress fiber was affected by the rigidity of the substrate. With 0.1 and 1 μg/μL, the cells on both soft and rigid surfaces displayed disrupted actin fibers, especially around their periphery where the MNPs@SiO_2_(RITC) accumulated. It was observed that a greater number of MNPs@SiO_2_(RITC) was taken in the cells on the rigid surfaces, compared to those on the soft surfaces (Additional file 1: Fig. S1). A similar report was found elsewhere [38].

### Effect of MNPs@SiO_2_(RITC) on filopodia formation of cells on soft and rigid surfaces

**Fig. 5:**
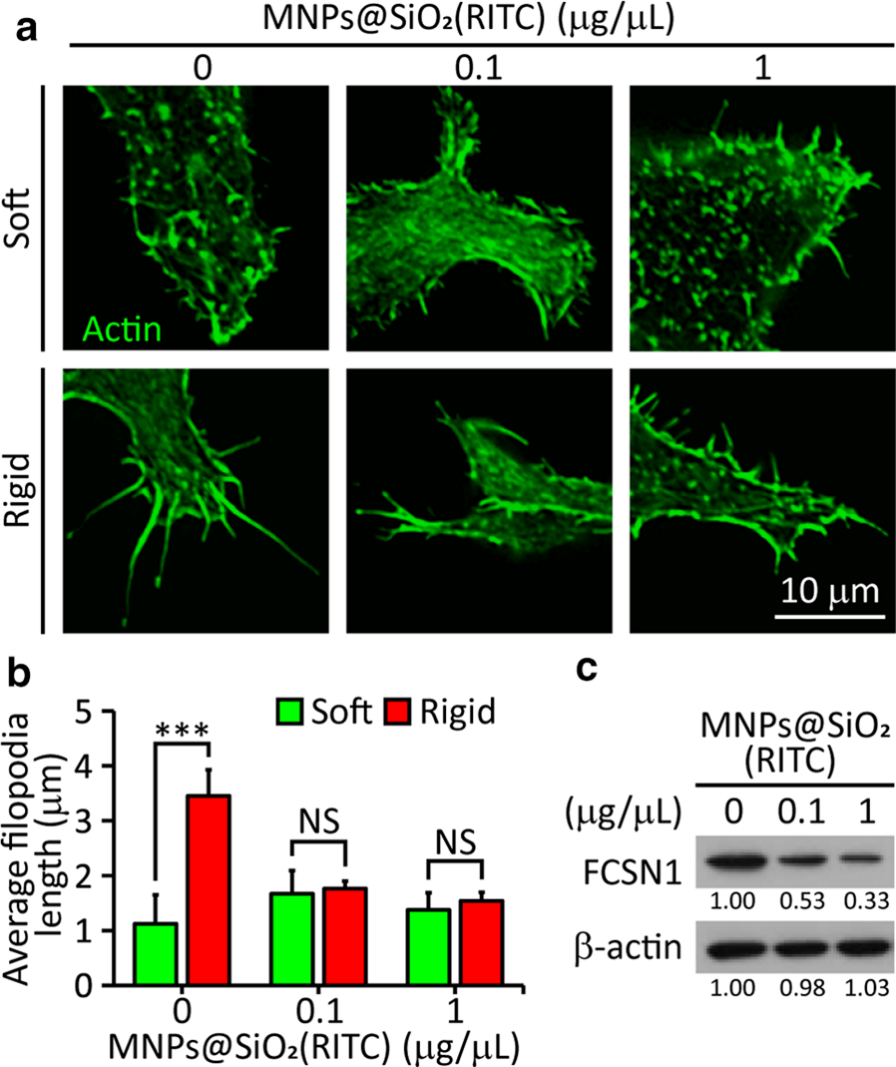
Filopodia formation of HEK293 cells on soft (5 kPa) and rigid (2 MPa) surfaces after treatment with different concentrations (0–1 μg/μL) of MNPs@SiO_2_(RITC). **a** Fluorescence images of cells with actin (green). **b** The length of filopodia (n = 323 in five cells). *** P < 0.001, NS: not significant. Student’s t-test. **c** Immunoblotting analysis of FCSN1

Filopodia are known to probe the rigidity of the ECM before cell spreading [33]. To assess whether MNPs@SiO_2_(RITC) treatment inhibits filopodia formation in HEK293 cells, the cells were incubated on soft (5 kPa) or rigid (2 MPa) surfaces with or without MNPs@SiO_2_(RITC) for 12 h, and the length of their filopodia was measured. The untreated cells on the rigid surface displayed filopodia longer than 3 μm, while those on the soft surface displayed short filopodia (about 1 μm) (Fig. 5a, b). These results indicate that the length of the filopodia is greatly affected by substrate rigidity, which is similar to previous reports [23]. At 0.1 and 1 μg/μL, cells on both soft and rigid surfaces displayed short filopodia, indicating the inhibition of filopodia formation (Fig. 5a, b). The result was supported by the western blot showing that the protein level of fascin (FCSN1), an actin bundling protein in filopodia, which decreased when cells were treated with MNPs@SiO_2_(RITC) at both 0.1 and 1 μg/μL concentrations (Fig. 5c). Taken together, these results suggest that cells lose their ability to sense substrate rigidity even at 0.1 μg/μL of MNPs@SiO_2_(RITC).

### Effect of MNPs@SiO_2_(RITC) on FA formation on soft and rigid surfaces

**Fig. 6:**
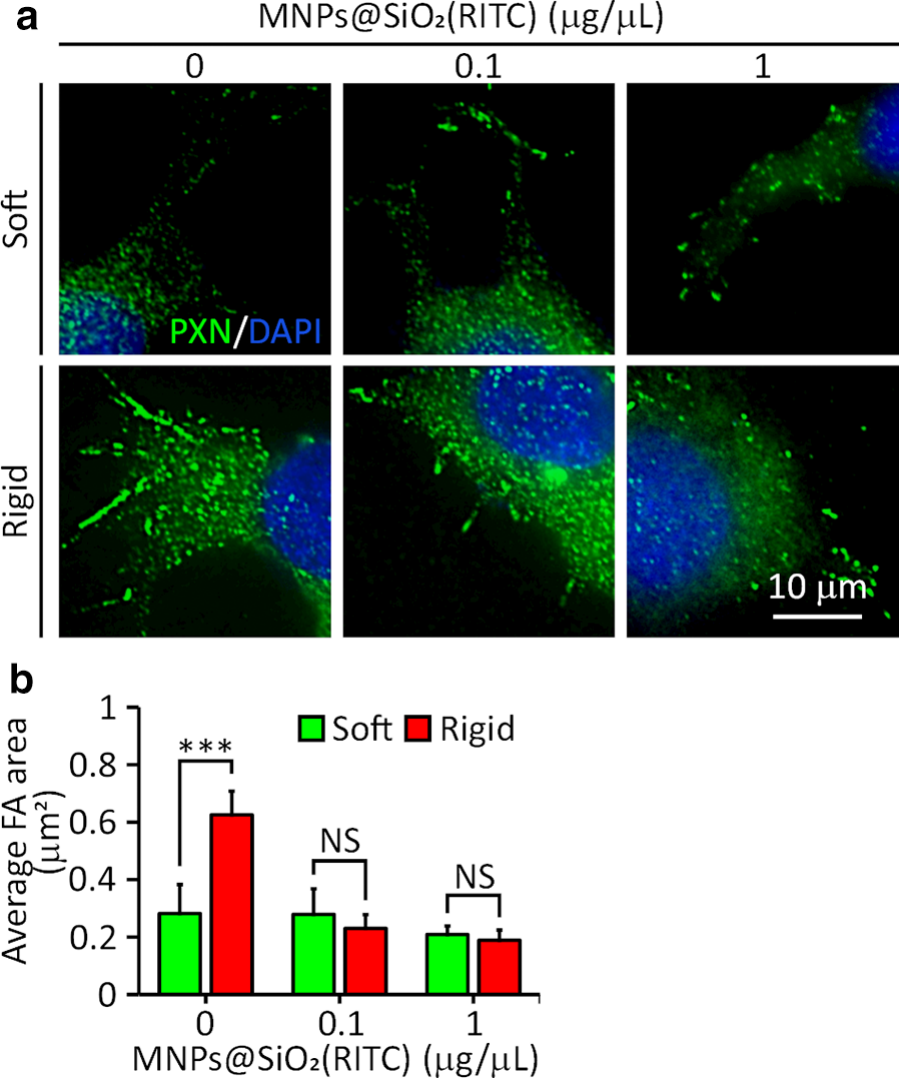
FA area of HEK293 cells on soft (5 kPa) and rigid (2 MPa) surfaces after treatment with different concentrations (0, 0.1, or 1 μg/μL) of MNPs@SiO_2_(RITC). **a** Fluorescence images of cells with anti-PXN primary antibody (green) and DAPI (blue) staining. **b** FA area. (n = 60 adhesions in seven cells). *** P < 0.001. NS: not significant. Student’s t-test

FAs are known to transmit signals of surface rigidity to the cell [39]. To assess whether MNPs@SiO_2_(RITC) inhibit FA formation in HEK293 cells, the FA area of MNPs@SiO_2_(RITC)-treated and untreated cells on both soft and rigid surfaces was measured after staining with an antibody against PXN, as a marker for FA [40]. Untreated cells formed larger FAs (0.6 μm^2^ on average) on rigid surfaces than on soft surfaces (0.28 μm^2^) (Fig. 6a, b). There was no difference in the FA area between the cells on both surfaces when they were treated with MNPs@SiO_2_(RITC) at 0.1 or 1 μg/μL (~ 0.3 μm^2^). These results showed that FA formation was inhibited even at 0.1 μg/μL of MNPs@SiO_2_(RITC).

### Effect of MNPs@SiO_2_(RITC) on local contractions of cells on pillars

**Fig. 7:**
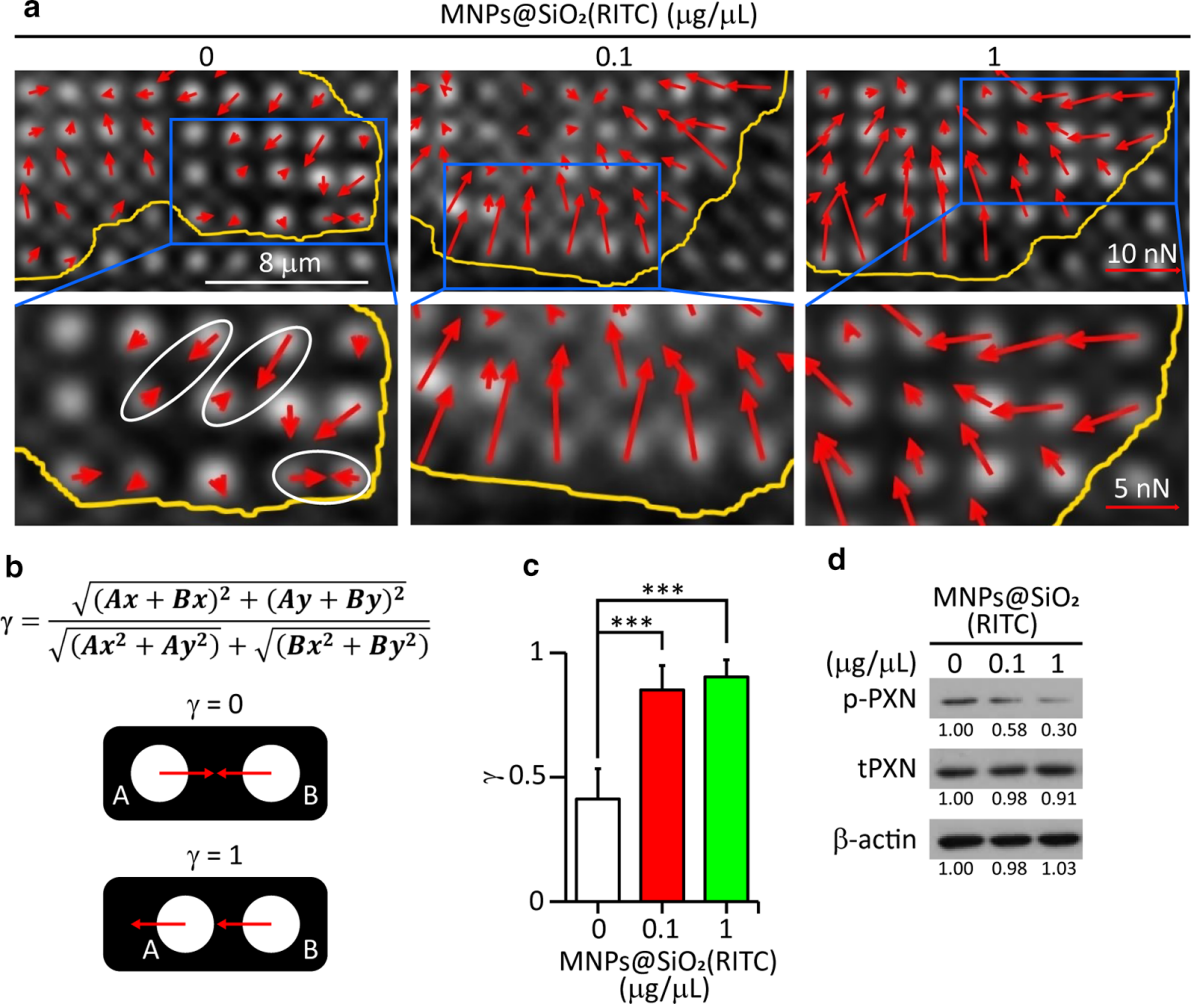
Spatial force distribution and directionality parameter. **a** Spatial force distribution (red arrows) at the edge of the cell. The yellow line indicates the approximate cell boundary. The direction and length of red arrows indicate the magnitude and direction of TF vectors. **b** Schematic of the directionality parameter (γ) and pillar forces. **c** Directionality parameter γ for areas of 34.5 μm^2^ on the edges of untreated or treated with MNPs@SiO_2_(RITC) at 0.1 µg or 1 µg/µL. (n = 20 frames of two cells for each sample). *** P < 0.001. Student’s t-test. A value of γ = 1 means that parallel forces are exerted on submicron pillars while γ = 0 means that locally balanced forces are exerted on the pillars. More details on this can be found in the Materials and Methods section. Student’s t-test. **d** Immunoblotting analysis of phosphorylated and total PXN. p-, phosphorylated protein; t, total protein. β-actin is used as an internal control

Cell rigidity sensing can be assessed by observing local contractions of neighboring submicron pillars toward each other at the edge of cells during the initial phase (~ 30 min) of cell spreading [36]. To assess the effect of MNPs@SiO_2_(RITC) on rigidity sensing, cells pre-treated with MNPs@SiO_2_(RITC) at different concentrations (0, 0.1, or 1 µg/µL) were seeded onto pillars coated with fibronectin, and images of pillars within a defined region (34.5 µm^2^) at the edge of cells were taken every 30 s for 30 min. In the untreated cells, neighboring pillars were deflected toward each other (Fig. 7a), which were not observed in the treated cells with MNPs@SiO_2_(RITC) at 0.1 or 1 µg/µL (Fig. 7a). The results were quantified by computing a directionality parameter, γ, defined as the magnitude of the sum of the force vectors found in an area of 34.5 μm^2^ at the cell periphery divided by the sum of their magnitudes [36] (Fig. 7b). The γ of the untreated cells was 0.41 ± 0.12 (mean ± SD, image number = 20), while the γ of the cells treated with MNPS@SiO_2_(RITC) at 0.1 or 1 µg/µL was 0.85 ± 0.1 or 0.9 ± 0.07 (Fig. 7c), respectively. Our results suggest that the rigidity sensing of cells is impaired by the treatment of MNPs@SiO_2_(RITC).

Local contractions in cells are driven by a sarcomere-like contractile unit consisting of several cytoskeletal components [41–43]. To understand how the treatment of MNPs@SiO_2_(RITC) at 0.1 or 1 µg/µL inhibited the rigidity sensing of the cells, immunoblotting of cytoskeletal proteins (MYL2 and PXN) and kinases (Src and FAK) regulating rigidity sensing was performed. The ratios of the phosphorylated protein level to the respective total protein level (Src, FAK, and MYL2) were lower for cells treated with 1 μg/μL MNPs@SiO_2_(RITC) than for untreated cells and cells treated with 0.1 μg/μL MNPs@SiO_2_(RITC) (Additional file 1: Fig. S2), and there was no clear difference between the phosphorylation of the proteins of the untreated cells and cells treated with 0.1 μg/μL MNPs@SiO_2_(RITC). A similar result was found in our previous study [23]. Previously, the effect of MNPs@SiO_2_(RITC) on the phosphorylation of PXN, which is involved in FA dynamics and rigidity sensing [23], was not studied. The ratio of phosphorylated PXN to total PXN decreased when the cells were treated with MNPs@SiO_2_(RITC) even at 0.1 μg/μL (Fig. 7d). These results indicate that the cytoskeletal proteins, including PXN and kinases, were all affected at 1 μg/μL, while only PXN was affected at 0.1 μg/μL. This result showed that the phosphorylation of PXN is a good indicator for evaluating the effect of low concentrations of MNPs@SiO_2_(RITC) on rigidity sensing.

## Discussion

Previously, the toxic effects of MNPs@SiO_2_(RITC), like damaging the cell membrane and the cytoskeleton and depleting ATP, were observed at a concentration of 1 μg/μL, but its effects at a lower concentration (0.1 μg/μL) have not yet been reported. Our study demonstrated that MNPs@SiO_2_(RITC) at a concentration of 0.1 μg/μL impaired rigidity sensing, which is necessary for cells to translate the mechanical properties of the ECM into biochemical signals that regulate the cell's genes and proteins [25–27]. To our knowledge, no study has been reported describing the effect of NPs on rigidity sensing in cells.

Impairment of rigidity sensing in cells was revealed by alterations in cell morphology (Fig. 3) and the formation of FA (Fig. 6) on soft and rigid surfaces. The untreated cells were polarized only on the rigid surfaces (Fig. 3). A similar result was observed in human foreskin fibroblast cells [41]. However, the treated cells were not polarized on the rigid surfaces (Fig. 3), which was supported by the shrinkage of the treated cells on another rigid surface (glass) (Fig. 2a, c). These inhibitory effects of MNPs@SiO_2_(RITC) can be explained by the disruption of actin cytoskeleton and defects in cytoskeletal proteins related to rigidity sensing (Figs. 4 and 7, Additional file 1: Fig. S2) [37, 41].

The effect of MNPs@SiO_2_(RITC) on rigidity sensing was revealed by alterations in the size of FA on the soft and rigid surfaces (Fig. 6). With the treatment, the FA area of the cells on the rigid surfaces decreased, resulting in no difference in the area between those on the soft and rigid surface. The impairment of rigidity sensing was confirmed by the relatively shorter filopodia lengths in the treated cells than in the untreated cells (Fig. 5a, b), consistent with the decrease in the expression of FSCN1 (Fig. 5c). The impairment of rigidity sensing by MNPs@SiO_2_(RITC) was quantified by the measurement of local contractions in the elastomeric pillars. With the treatment of MNPs@SiO_2_(RITC), locally contracted pillars disappeared, indicating that the rigidity sensing of the treated cells was impaired (Fig. 7a, c). Taken together, it is suggested that the treated cells cannot discern the rigidity difference between soft and rigid surfaces.

FAK activity and PXN phosphorylation regulate FA traction dynamics, which are required for a cell to discern rigidity differences at high spatial resolutions [44]. Previously, we reported that the phosphorylation of FAK and Src, markers for the formation of FA, decreased in bone marrow-derived mesenchymal stem cells when treated with MNPs@SiO_2_(RITC) at 1 µg/µL. In this study, we reported that in addition to the phosphorylation of FAK and Src (Additional file 1: Fig. S2) the phosphorylation of PXN decreased even at 0.1 µg/µL (Fig. 7d). It was reported that the ROS generated by the NPs plays a role in reducing the phosphorylation of cytoskeletal proteins including the phosphorylation of PXN [45]. This result suggests that the reduction of PXN phosphorylation may disturb FA traction dynamics, which is required for rigidity sensing [44].

NPs can interact with the cytoskeleton directly during internalization (endocytosis) through the cell membrane [46] or after internalization through the production of ROS [47]. It was reported that the excess ROS produced by MNPs@SiO_2_(RITC) decreased the expression of the main FA proteins such as Src and FAK through lipid peroxidation [23]. Since NPs could interact with the cytoskeleton through integrin signaling during cell attachment and rigidity sensing, it is suggested that cytoskeletal proteins related to rigidity sensing were affected by the excess ROS produced by MNPs@SiO_2_(RITC), even at 0.1 µg/µL. This study indicates that NPs at low concentrations may impair the rigidity sensing of cells, thereby affecting subsequent cell behaviors and fates.

## Conclusion

This study suggests that a low concentration of MNPs@SiO_2_(RITC) may impair the rigidity sensing of the cell and could further affect cellular function, such as cell spreading and adhesion formation. By affecting cell spreading, NPs could affect 3D shape of cells and their physiological functions of normal epithelial cells. On the other side, NPs can be used to reduce tumor dissemination by inhibiting filopodia formation, which is related to cancer invasion. In general, this study implies that mechanobiological methods such as rigidity sensing are more sensitive than conventional methods to study the effect of MNPs on the cells before using them for biomedical purposes.

## Materials and methods

### Preparation of of MNPs@SiO_2_(RITC)

MNPs@SiO_2_(RITC) were prepared with a ~ 9 nm cobalt ferrite core (CoFe_2_O_3_) chemically bonded to rhodamine isothiocyanate (RITC) dye and coated by a silica shell [15]. MNPs@SiO_2_(RITC) are 50 nm in diameter and are known to have zeta potentials between − 40 to − 30 mV [15, 48].

### Cell culture

Human embryonic kidney 293 (HEK293) cells were obtained from American Type Culture Collection (ATCC, Manassas, VA, USA). Briefly, cells were cultured in Dulbecco’s high-glucose modified Eagle’s medium (Gibco, Carlsbad, CA, USA) supplemented with 10% fetal bovine serum (Gibco), 100 units/mL penicillin, and 100 µg/mL streptomycin (Gibco) and incubated humidified chamber at 37 °C in a 5% CO_2_ atmosphere.

### Treatment of cells with MNPs@SiO_2_(RITC)

The dosage used in this study was determined by treating HEK293 cells with MNPs@SiO_2_(RITC) at concentrations ranging from 0.01 to 2 µg/µL for 12 h and calculating their uptake efficiencies [24]. The uptake efficiency of MNPs@SiO_2_(RITC) was known to be plateaued at 1 µg/µL [24]. The optimal concentration of MNPs@SiO_2_(RITC) was 0.1 µg/µL for in vitro use and as a magnetic resonance imaging contrast [22]. Thus, MNPs@SiO_2_(RITC) at 0.1 µg/µL and 1 µg/µL were used in the present study.

### Immunocytochemistry and cell area analysis

Cells were seeded on cover slips and incubated for 6 h at 37 °C and 5% CO_2_. Then, the attached cells on the coverslips were treated with 0.1 µg/µL or 1.0 µg/µL of MNPs@SiO_2_(RITC) for 12 h at 37 °C and 5% CO_2_. The cells were then fixed in Cytofix buffer (BD Bioscience, San Jose, CA, USA) for 30 min at 4 ℃. To reduce non-specific binding, the cover slips were blocked with phosphate-buffered saline (PBS, pH 7.4) containing 2% bovine serum albumin (BSA) and 0.1% Triton-X100 (Sigma-Aldrich, St. Louis, MO, USA). For actin labeling, cells were incubated with Alexa Fluor 488-conjugated phalloidin (Molecular Probe, Eugene, OR, USA), (1:200) diluted in PBS, for 1 h at room temperature (RT). For nuclear labeling, cells were washed three times with PBS containing 0.1% Triton-X100 and incubated with PBS containing 10 µg/mL of Hoechst 33,342 (Thermo Fisher Scientific, Waltham, MA, USA) for 10 min at RT. After washing three times with PBS, cover slips were mounted onto slides using Prolong Gold Antifade mounting medium (Molecular Probe). Fluorescence images were acquired by confocal laser scanning microscopy (CLSM) with a slide scanner (Axioscan. Z1, Carl Zeiss Microscopy GmbH, Jena, Germany). Attached cell areas were determined using ImageJ (NIH, Bethesda, MD, USA).

### Preparation of soft and rigid flat PDMS surfaces

Surfaces were prepared using a Sylgard Silicone Elastomer Kit (Dow Corning, Cortland, NY, USA). The silicone elastomer base was mixed with a curing agent, degassed for 30 min, and spin-coated on a cover glass at 2,000 rpm for 2 min to obtain a PDMS layer 35 ± 5 μm thick. Then, the elastomer was crosslinked at 70 °C for 4 h. Surfaces with Young's moduli of 5 kPa (soft) and 2 MPa (rigid) were prepared by changing the ratio of elastomer base to curing agent to 75:1 and 10:1, respectively. Coverslips with a layer of PDMS were functionalized with 20 μg/mL fibronectin (Sigma-Aldrich) at 4 °C overnight. Prior to cell seeding, coverslips with PDMS layers were washed with PBS and growth medium. Then cells were seeded and treated with MNPs@SiO_2_(RITC) at 0.1 and 1 μg/μL for 12 h.

### Cell morphological analysis on soft and rigid flat PDMS surfaces

Untreated and MNPs@SiO_2_(RITC)-treated cells were incubated on soft or rigid surfaces for 12 h and fixed with 4% paraformaldehyde in PBS for 15 min to study the spreading area, aspect ratio, and filopodia structure of the cells. They were then permeabilized with 0.1% Triton X-100 in PBS for 15 min. Next, the actin structures of the cells were labeled with Alexa Flour 488 phalloidin (Thermo Fisher Scientific) (1:2500) in PBS for 30 min at RT. The cell nuclei were then labeled with 0.2 mg/mL of 4′,6-diamidino-2-phenylindole (DAPI) (Sigma-Aldrich) (1:10,000) in PBS for 15 min at RT. Finally, the stained cells were imaged using a DeltaVision microscope (GE Healthcare, Chicago, IL, USA) equipped with a CoolSnap HQ2 camera (Photometrics, Tucson, AZ, USA). The cell spreading area and cell aspect ratio were measured using FIJI ImageJ (NIH), and the filopodia of cells were analyzed using a FIJI ImageJ plug-in called FiloQuant [49].

### FA size analysis on soft and rigid flat PDMS surfaces

Cells were fixed and permeabilized for FA analysis as previously described. The cells were then blocked with 1% BSA (Gibco) for 1 h. The cells were subsequently treated with anti-paxillin primary antibody (ab32084, Abcam, London, UK) 1:500 in 1% BSA for 1 h at RT, and the cell nuclei were labeled with DAPI for 15 min. The cells were then imaged with a DeltaVision microscope (GE Healthcare). Finally, the FA area of the cells was analyzed using FIJI ImageJ.

### Western blotting

For analysis of cytoskeletal proteins, untreated and MNPs@SiO_2_(RITC)-treated cells were lysed in a protease inhibitor cocktail (Sigma-Aldrich) containing radioimmunoprecipitation assay (RIPA) buffer (Sigma-Aldrich), and total protein concentrations were determined by the bicinchoninic acid kit (Bio-Rad, Hercules, CA, USA). Proteins were separated using 10% SDS-PAGE and transferred onto nitrocellulose membranes. The membranes were then washed three times with 0.1% Tween-20 (Sigma-Aldrich) in tris-tween buffered saline (T-TBS) and blocked with 5% non-fat milk/T-TBS. The membranes were incubated with mouse monoclonal antibodies against phosphorylated Src (p-Src), total Src (tSrc), phosphorylated FAK (p-FAK), total FAK (tFAK), phosphorylated MLY2 (p-MLY2), total (tMLY2), phosphorylated paxillin (p-PXN), total PXN (tPXN), β-actin (1:1000; Santa Cruz Biotechnology, Dallas, TX, USA), and anti-rabbit FSCS1 (ab126772) (1:10,000; Abcam) as primary antibodies. After washing with T-TBS three times, the membranes were incubated with mouse Ig kappa binding protein conjugated with horseradish peroxidase (1:1000; Santa Cruz Biotechnology). Blots were developed using an enhanced chemiluminescence solution (Thermo Fisher Scientific), and luminescence was captured on medical blue X-ray films (Agfa, Mortsel, Belgium) in a dark room.

### Pillar fabrication and pillar force analysis

Photolithography using reactive ion etching was used to fabricate a linear array of holes (0.9 µm diameter, 2 µm depth, 1.8 µm distance between holes) in a 5-inch silicon wafer. The PDMS pillar array (2 μm in height, 0.9 μm diameter, 1.8 μm center to center distance, *k* = 24.21 nN/μm) was made from the Sylgard Silicone Elastomer Kit. The silicone elastomer base was mixed with the curing agent in a ratio of 10:1 and then degassed for 15 min to remove air bubbles. The mixed elastomer was then spin-coated on the mold at 1000 rpm for 1 min and degassed for 15 min. The coated mold was then cured at 80 °C for 3 h. The pillar array was removed from the silicon mold while immersed in 99.5% isopropanol. The bending stiffness k of the column was calculated using the Euler Bernoulli beam theory:

$$k=\frac{3}{64} \pi E\frac{{D}^{4}}{{L}^{3}}$$

where D and L are the diameter and length of the pillar, respectively, and E is the Young’s modulus of the PDMS (2 MPa). In this study, the stiffness (*k*) of the pillar was 24.21 nN/μm.

For cellular traction force measurements, cells were incubated with MNPs@SiO_2_(RITC) at 0.1 or 1 µg/µL for 12 h at 37 °C, 5% CO_2_. Then they were washed three times with PBS, detached from the dish using trypsin (Gibco), and collected. Next, they were seeded onto pillars previously coated with 10 μg/mL fibronectin and incubated for 1 h at 37 °C, 5% CO_2_. Images of cells on the pillars were taken using a DeltaVision (GE Healthcare) microscope equipped with a CoolSnap HQ^2^ (Photometrics) camera under a live cell chamber (Live Cell Instrument, Seoul, Korea) at 37 °C and 5% CO_2_. Pillar deflection was measured using the Pillar Tracker 1.1.3 version obtained from the Mechanobiology Institute in Singapore. Pillar Tracker applies a reconstruction algorithm to create a complete grid for measuring pillar deflection. The pillars outside the cell were used as a reference for the zero-force position. The deflection value was then multiplied by the bending stiffness of the pillar to determine its traction force. Local contraction was quantified using the directional parameter (γ) during the initial phase of cell spreading (30 min) at the leading edge of the cell (an area of 34.5 µm^2^) [36]. The directional parameter (γ) was calculated as the sum of the pillar force vectors divided by the sum of their magnitudes. For two equal neighboring pillar forces A and B, γ will be:

$$\gamma = \frac{{\sqrt {\left( {Ax + Bx} \right)^{2} + \left( {Ay + By} \right)^{2} } }}{{\sqrt {\left( {Ax^{2} + Ay^{2} } \right)} + \sqrt {\left( {Bx^{2} + By^{2} } \right)} }}$$

For this purpose, cell images taken within 30 min of cell surface contact were used for γ calculation. Average γ values for each type of cell were obtained by calculating 20 frames of two different cells.

### Statistical analysis and error correction

Data are represented as the mean ± standard error of the mean of the samples in a group. Student’s t-test was used to compare 0.1 or 1 μg/μL MNPs@SiO_2_(RITC)-treated samples with untreated samples. A p-value of less than 0.05 was considered significant (*P < 0.05, **P < 0.01, ***P < 0.001). In the experiments using pillars, errors of the pillar deflections were corrected by reducing the average pillar deflection of pillars outside the cell.

## Supplementary information

**Additional file 1:** **Fig S1. **MNPs@SiO_2_(RITC) intensity per μm^2^ area of the cell on soft and rigid PDMS surfaces with MNPs@SiO_2_(RITC) at different concentrations (0-1 µg/μL) for 12 h. (n=5 cells), NS (not significant) P >0.05, *** P <0.001 Student’s t-test. **Fig S2. **Immunoblotting analysis of phosphorylated and total Src, FAK and MYL2. p-, phosphorylated protein; t, total protein. β-actin is used as an internal control.

## Data Availability

Data sharing is not applicable to this article as no datasets were generated or analyzed during the current study.

## Abbreviations

NPs: Nanoparticles
MNPs@SiO_2_(RITC): Silica-coated magnetic nanoparticles incorporating rhodamine B isothiocyanate
ROS: Reactive oxygen species
MNPs: Magnetic nanoparticles
ECM: Extracellular matrix
PDMS: Polydimethylsiloxane
HEK293: Human embryonic kidney cells
FA: Focal adhesion
PBS: Phosphate-buffered saline
BSA: Bovine serum albumin
RT: Room temperature
DAPI: 4′,6-Diamidino-2-phenylindole
RIPA: Radioimmunoprecipitation assay
T-TBS: Tris-tween buffered saline
Src: Non-receptor tyrosine kinase
FAK: Focal adhesion kinase
MLY2: Myosin light chain II
PXN: Paxillin
FCSN1: Fascin
ATP: Adenosine triphosphate
