# Supplementary material for: Effect of silica-coated magnetic nanoparticles on rigidity sensing of human embryonic kidney cells
Source: J Nanobiotechnology. 2020 Nov 18;18:170. doi: 10.1186/s12951-020-00730-2 (PMC7672867; doi:10.1186/s12951-020-00730-2)
Supplement: Supplementary file 1 — Additional file 1: Fig S1. MNPs@SiO2(RITC) intensity per μm2 area of the cell on soft and rigid PDMS surfaces with MNPs@SiO2(RITC) at different concentrations (0-1 µg/μL) for 12 h. (n=5 cells), NS (not significant) P >0.05, *** P <0.001 Student’s t-test. Fig S2. Immunoblotting analysis of phosphorylated and total Src, FAK and MYL2. p-, phosphorylated protein; t, total protein. β-actin is used as an internal control. [file 12951_2020_730_MOESM1_ESM.docx]

**Additional file 1**

**Effect of silica-coated magnetic nanoparticles on rigidity sensing of human embryonic kidney cells**

Abdurazak Aman Ketebo^1+^, Tae Hwan Shin^2+^, Myeongjun Jun^1^, Gwang Lee^2,*^ and Sungsu Park^1,*^

^1^School of Mechanical Engineering, Sungkyunkwan University, Suwon 16419, Korea

^2^Department of Physiology, Ajou University School of Medicine, Suwon 16499, Korea

^+^Each author contributed equally to this work

^*^**Corresponding authors:** G. Lee, Department of Physiology, Ajou University School of Medicine, Suwon 16499, Korea. Tel: +82-31-219-4554. E-mail: glee@ajou.ac.kr/S. Park, School of Mechanical Engineering, Sungkyunkwan University, Suwon, 16419, Korea. Tel: +82-31-290-7431. E-mail: nanopark@skku.edu


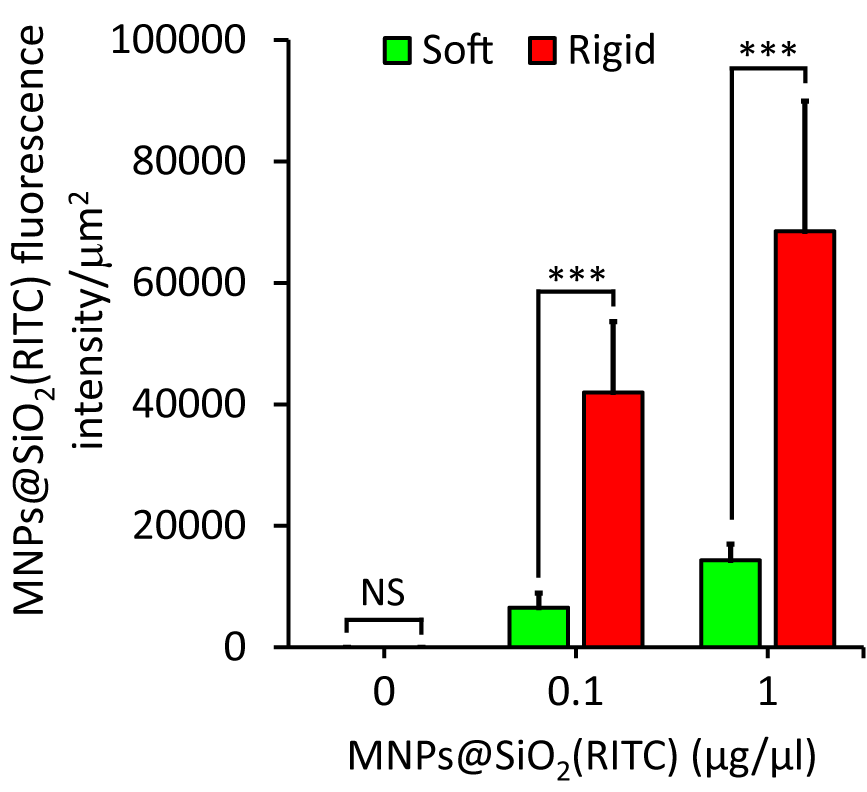
**Fig S1.** MNPs@SiO_2_(RITC) intensity per μm^2^ area of the cell on soft and rigid PDMS surfaces with MNPs@SiO_2_(RITC) at different concentrations (0-1 µg/μL) for 12 h. (n=5 cells), NS (not significant) P >0.05, *** P <0.001 Student’s t-test.

**
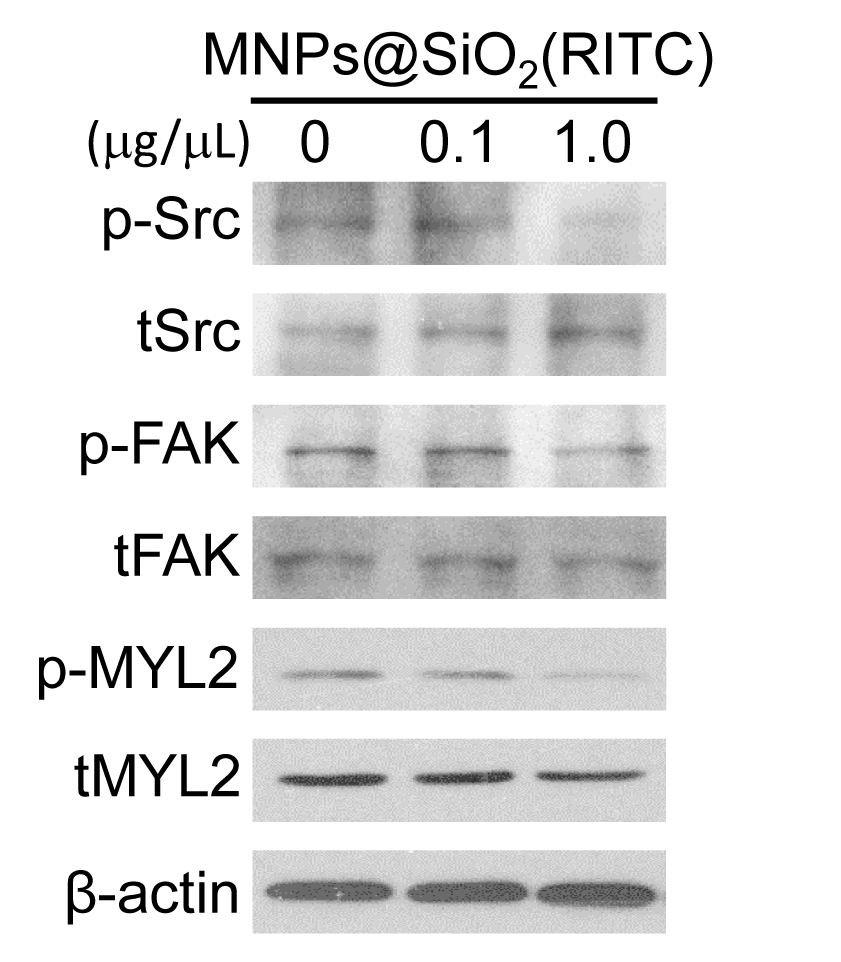
Fig S2.** Immunoblotting analysis of phosphorylated and total Src, FAK and MYL2. p-, phosphorylated protein; t, total protein. β-actin is used as an internal control.
